# Acute Deterioration of Pulmonary Arterial Hypertension (PAH) in a Patient with Neurofibromatosis Type 1 (NF1)

**DOI:** 10.1155/2019/2987461

**Published:** 2019-07-22

**Authors:** Seiya Tanaka, Fuko Kawahara, Taro Miyamoto, Satoshi Tsurusaki, Yoshihito Sanuki, Kiyoshi Ozumi, Takashi Harada, Hiromi Tasaki

**Affiliations:** ^1^Department of Cardiovascular Medicine, Kitakyushu Municipal Yahata Hospital, 2-6-2 Okura, Yahatahigashi-ku, Kitakyushu 805-8534, Japan; ^2^Department of Internal Medicine, Kitakyushu Municipal Yahata Hospital, 2-6-2 Okura, Yahatahigashi-ku, Kitakyushu 805-8534, Japan

## Abstract

A 56-year-old woman was diagnosed as having chronic obstructive pulmonary disease with heavy smoking. Mild pulmonary hypertension (mean pulmonary arterial pressure: 31 mmHg) was detected at the first visit. She was diagnosed with pulmonary hypertension due to pulmonary disease and medicated only with bronchodilators. Simultaneous, multiple freckling in the trunk of her body and café au lait macules in her back with some cutaneous neurofibromas were also detected. A plastic surgeon removed one of the neurofibromas and pathologically diagnosed it as neurofibromatosis type 1 (NF1). We finally rediagnosed her with pulmonary hypertension with unclear and/or multifactorial factors when she deteriorated 1 year after being treated only with bronchodilators. We then administrated upfront combination therapy with macitentan and tadalafil. Mean pulmonary arterial pressure rapidly improved. *Learning Objective*. Pulmonary arterial hypertension (PAH) in neurofibromatosis type 1 (NF1) can occur due to lung disease or due to certain involvement of pulmonary arteries, or a combination of both. Increased awareness of PAH in NF1 is very important for patients survival. The current therapeutic strategy is almost identical to that of idiopathic PAH; however, there is no clinical evidence. Insights gained from clinical experiences should help identify promising novel therapeutic approaches in NF1-PAH.

## 1. Introduction

Neurofibromatosis type 1 (NF1) is a relatively common genetic disorder with an incidence of approximately 1 in 4000 live births. NF1 is characterized by cutaneous neurofibromatosis and café au lait spots, and they are inherited in an autosomal dominant pattern [1, 2]. Arterial vasculopathies are well-recognized manifestations of this disease and one of the most important causes of a poor prognosis in patients in NF1. Pulmonary arterial hypertension (PAH) associated with NF1 is included in the current classification of pulmonary hypertension in the group with unclear and/or multifactorial mechanisms [3–5]. The present case was initially treated for pulmonary hypertension with chronic obstructive pulmonary disease (COPD), which belongs to group 3 of the clinical classification of pulmonary hypertension [6]. In recognition of NF1 as a distinct cause of PAH, we propose that it should be termed NF1-associated PAH.

## 2. Case Presentation

**Figure 1:**
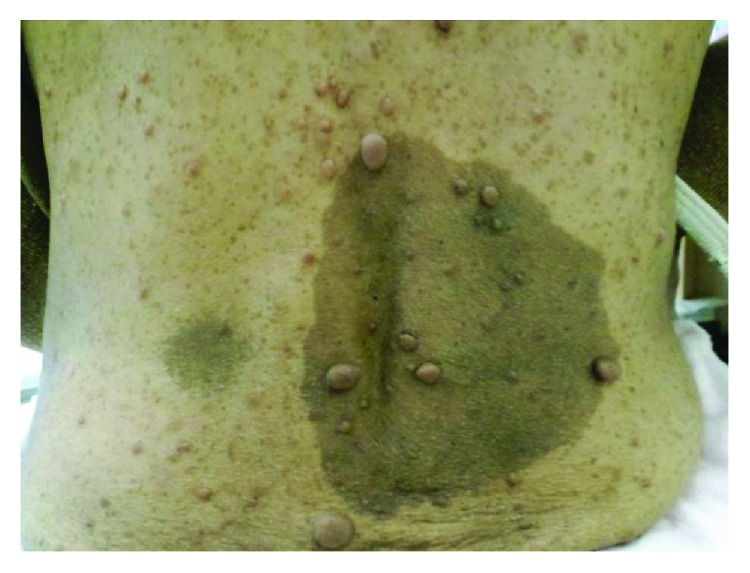
Patient with multiple cutaneous neurofibromatosis and café au lait macules.

**Figure 2:**
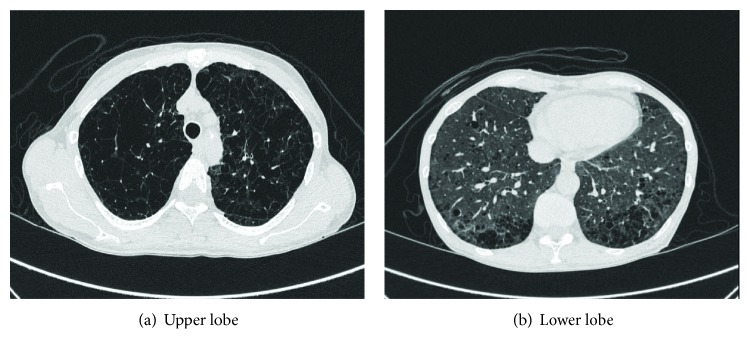
High resolution CT of the chest shows emphysema of the upper lobe (a) and bilateral mosaic pattern of the lower lobe (b).

A 54-year-old woman was diagnosed with COPD. Her blood pressure was 104/56 mmHg, pulse was 87 beats/min, and respiratory rate was 22 breaths/min. She was in WHO functional class II. She had multiple both cutaneous neurofibromas and café au lait macules and freckling in her back and the axilla (Figure 1). She was referred to the Department of Respiratory Medicine of our hospital in 2016. Right heart cardiac catheterization (RHC) showed mild pulmonary arterial pressure (PAP) of 49/13 (mean: 31 mmHg), pulmonary capillary wedge pressure of 7 mmHg, and pulmonary vascular resistance (PVR) of 476 dyne·s·cm^−5^. Cardiac output (CO) was 4.0 L/min and the cardiac index was 2.9 L/min/m^2^. High-resolution computed tomography (HRCT) showed markedly enlarged central pulmonary arteries, upper-lobe centrilobular emphysema with multiple bullae, and bilateral ground-glass nodules with multiple cystic lesions in the lower lobe (Figure 2). A ^99m^Tc macroaggregated albumin lung perfusion scan showed perfusion defects of bilateral upper lobes, and these defects matched emphysematous lesions of her lung. An obstructive pattern was observed in a respiratory functional test as follows: forced expiratory volume in 1 s, 1.86 L (83% of predicted); forced vital capacity 2.88 (105.5% of predicted); and diffusion capacity of lung for carbon monoxide 3.6 ml/min/mmHg (23.9% of predicted). The results of the tests for rheumatoid factor, antinuclear antibody, anti-scleroderma antibody, anti-smith antibody, anti-ribonucleoprotein antibody, and anti-Sjogren's syndrome A/Sjogren's syndrome B antibodies were all negative. The patient denied any history of diet drug use, human immunodeficiency virus risk factors, obstructive sleep apnea symptoms, or rheumatological symptoms.

At initial assessment, she was diagnosed with pulmonary hypertension in the group of pulmonary hypertension due to lung disease. A respiratory physician initiated full medications of bronchodilators and home oxygen therapy to her. She did not have a family history of NF1 or PAH. She had already noticed neurofibromas when she was in elementary school. She did not have any symptoms associated with the neurofibromas but had always wanted to remove some of them cosmetically. She consulted a plastic surgeon in our hospital for the first time. He removed two of her neurofibromas and pathologically diagnosed them as NF1.

**Figure 3:**
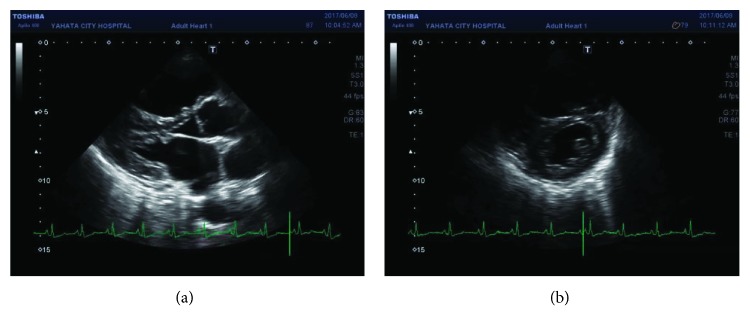
Transthoracic echocardiography shows that the left ventricle was compressed due to the enlargement of the right ventricle during end-diastole in deteriorating pulmonary hypertension, parasternal long-axis view (a), and parasternal short axis view (b).

One year after medicating with bronchodilators, her exertional dyspnea progressed to WHO functional class III. Echocardiography showed enlargement of the right ventricle with ventricular septal flattening during systole (Figure 3). RHC showed hemodynamic deterioration. PAP and PVR were increased to 81/26 (mean: 48 mmHg) and 1041 dyne·s·cm^−5^, respectively. CO was decreased to 2.92 L/min. HRCT showed no change in lung parenchymal involvement. A respiratory functional test also showed no change. These results suggested that COPD did not contribute to her deterioration. At this point, the patient's diagnosis was likely PAH associated with NF-1. Recent classification of pH places in group 5 which is systemic disorders with unclear and/or multifactorial mechanisms [6]. The treatment algorithm for group 1 pulmonary hypertension in the European Society of Cardiology and the European Respiratory Society guidelines was applied [7]. We chose the upfront combination therapy with macitentan 10 mg and tadalafil 40 mg because our patient was in WHO functional class III and the prognosis of NF-1 associated with PAH is extremely poor [3, 8]. These reports suggested that treatment of NF1 with PAH should be modeled on treatment of group 1 PAH. After 1 month, our patient's symptom was relieved to WHO functional class II. RHC showed hemodynamic improvement. PAP and PVR were decreased to 49/17 (mean: 30 mmHg) and 356 dyne·s·cm^−5^, respectively. Currently, 1 year after upfront combination therapy, the patient's condition is stable.

## 3. Discussion

NF1 is an autosomal dominant condition that is caused by heterozygous mutations of the *NF1* gene, which is located on chromosome 17 [2]. NF1 has approximately 100% penetrance and variable phenotype expression, and 50% of cases are sporadic. This disorder mainly involves the skin, peripheral nervous system (neurofibromatosis), and iris (Lisch nodules). Our patient met the diagnosis of NF1 because she had more than six café au lait macules of >15 mm in greatest diameter, multiple cutaneous neurofibromas, and axillary freckling [9].

The prognosis is generally good in most patients with NF1. However, the clinical course of NF1 is occasionally complicated by arterial vasculopathies, which involve the cerebral renal coronary and peripheral vascular beds. Pulmonary arteriopathy and secondary PAH with NF1 (NF1-PAH) are rare and their underlying mechanisms are unclear and multifactorial. This condition is classified as group 5 in an updated comprehensive version of the clinical classification of pulmonary hypertension [7]. To the best of our knowledge, only 23 cases of this association have been reported [3–5, 8]. NF1-PAH is an extremely severe complication of NF1 and is characterized by late-onset, female predominance, severe functional and hemodynamic impairment, a poor response to PAH-specific agents, and a poor outcome.

In our patient, a respiratory physician initially concluded that COPD was responsible for dyspnea or pulmonary hypertension. However, even under the full medication with bronchodilators and home oxygen therapy, her condition suddenly deteriorated without progression of lung parenchymal lesions. The etiological diagnosis of NF1-PAH is difficult in the presence of lung parenchymal lesions because lung abnormalities are variable in patients with NF1. A PFT invariably shows some obstructive, restrictive, or mixed patterns. Most of these patients have an impaired diffusion capacity, even with a normal lung volume, which suggests vascular involvement. Ventilation perfusion scans and chest HRCT showed bilateral filling defects and a mosaic pattern (representing irregular perfusion) in our patient, which also suggested vascular involvement as a primary cause of PAH. Although there are currently no specific therapeutic guidelines for NF1-PAH, optimal management requires the advice of an expert referral center [7]. Little is known about the pulmonary arteries in NF1-PAH. Plexogenic arteriopathy and complex plexiform lesions similar to those observed in idiopathic PAH have been observed in autopsy specimens in NF1-PAH [3]. This evidence suggests that treatment of NF1-PAH should be equivalent to treatment of group 1 PAH. We decided to use upfront combination therapy of an endothelin receptor antagonist and a phosphodiesterase type 5 inhibitor.

A recent report indicated that degenerated pulmonary vasculature in NF1-associated PAH is affected by NF1 vasculopathy [10]. Another report showed that regulation of Ras by NF1 plays a critical role in vascular smooth muscle proliferation [6]. Sorafenib is an oral inhibitor of multiple kinases, including Raf-1, which is the downstream target of Ras in the MAPK cascade, and may have a beneficial therapeutic effect in NF1-PAH.

## 4. Conclusion

Pulmonary hypertension in patients with NF1 can occur because of parenchymal lung disease, certain involvement of pulmonary arteries, or a combination of both. Recognizing this association is important because it implicates mutations in the *NF1* gene with the pathogenesis of pulmonary vasculopathy. Furthermore, early recognition is warranted because of rapid progression and poor prognosis.
